# Prevalence and risk factors of moderate to severe obstructive sleep apnea syndrome in insomnia sufferers: a study on 1311 subjects

**DOI:** 10.1186/s12931-017-0616-8

**Published:** 2017-07-06

**Authors:** Matthieu Hein, Jean-Pol Lanquart, Gwénolé Loas, Philippe Hubain, Paul Linkowski

**Affiliations:** Department of Psychiatry and Sleep Laboratory, Erasme Hospital, Université Libre de Bruxelles, ULB, Route de Lennik, 808, 1070 Anderlecht, Brussels Belgium

**Keywords:** Insomnia, Obstructive apnea syndrome, Prevalence, Risk factors

## Abstract

**Background:**

Several studies have investigated the prevalence and risk factors of insomnia in subjects with obstructive sleep apnea syndrome. However, few studies have investigated the prevalence and risk factors for obstructive sleep apnea syndrome in insomnia sufferers. Thus, the aim of this study was to examine the prevalence and risk factors of moderate to severe obstructive sleep apnea syndrome in a large sample of insomnia sufferers.

**Methods:**

Data from 1311 insomnia sufferers who were recruited from the research database of the sleep laboratory of the Erasme Hospital were analysed. An apnea-hypopnea index of ≥15 events per hour was used as the cut-off score for moderate to severe obstructive sleep apnea syndrome. Logistic regression analyses were conducted to examine clinical and demographic risk factors of moderate to severe obstructive sleep apnea syndrome in insomnia sufferers.

**Results:**

The prevalence of moderate to severe obstructive sleep apnea syndrome in our sample of insomnia sufferers was 13.88%. Multivariate logistic regression analysis revealed that male gender, snoring, excessive daytime sleepiness, lower maintenance insomnia complaint, presence of metabolic syndrome, age ≥ 50 & <65 years, age ≥ 65 years, BMI ≥ 25 & <30 kg/m^2^, BMI >30 kg/m^2^, and CRP >7 mg/L were significant risk factors of moderate to severe obstructive sleep apnea syndrome in insomnia sufferers.

**Conclusion:**

Moderate to severe obstructive sleep apnea syndrome is a common pathology in insomnia sufferers. The identification of these different risk factors advances a new perspective for more effective screening of moderate to severe obstructive sleep apnea syndrome in insomnia sufferers.

## Highlights

- The prevalence of moderate to severe OSA in this sample of insomnia sufferers from a general sleep laboratory is 13.88%.
- Male gender, age ≥ 50 & <65 years, age ≥ 65 years, BMI ≥25 & <30 kg/m^2^, BMI >30 kg/m^2^, snoring, presence of metabolic syndrome, excessive daytime sleepiness, lower maintenance insomnia complaints and higher plasma levels of CRP were risk factors for moderate to severe OSA in insomnia sufferers.
- These risk factors open up a new perspective for more effective screening of moderate to severe OSA in insomnia sufferers.

## Background

Obstructive sleep apnea syndrome (OSA) is characterized by repetitive episodes of upper airway obstruction that occur during sleep usually associated with a reduction in blood oxygen saturation [1]. The clinical manifestations of OSA include witnessed apneas, snoring, choking/gasping episodes, excessive daytime sleepiness, non-restorative sleep, nocturia, sleep fragmentation/sleep maintenance insomnia, long duration of sleep, morning headaches, loss of libido, irritability, and decreased concentration and memory [2]. Some of these symptoms are also present in insomnia sufferers, which may make diagnosis and management of OSA more difficult in these subjects [3]. Both insomnia and moderate to severe OSA (apnea-hypopnea index [AHI] ≥15/h) [4] are associated with a higher risk of cardiovascular morbidity and mortality [5, 6], which justifies the introduction of effective treatment [7].

The co-occurrence of insomnia and OSA is very common [8, 9]. Indeed, depending on the studies, the prevalence of insomnia complaint in OSA may reach 54.9% [10], whereas the prevalence of OSA (AHI ≥5/h) in insomnia sufferers may reach 67% [11]. Additionally, other studies have shown a 29% prevalence of moderate to severe OSA in insomnia sufferers [12, 13] over the age of 60 years in contrast to 1%-14% (9%-14% of men and 2%-7% of women) [14] in the general population and up to 18% in individuals over 40 years of age [15]. The physiopathology of the particular relationship between insomnia and OSA is still unknown. OSA could promote insomnia by developing psychophysiological conditioning in response to repeated awakenings with dysfunctional sleep behaviors as consequences [16]. Conversely, insomnia could aggravate OSA through sleep fragmentation and frequent phase changes leading to predominance of superficial NREM sleep [17], which could aggravate the instability of the upper airways [18]. Thus, it would be interesting to study both the prevalence and risks factors of moderate to severe OSA with a larger sample of insomnia sufferers to better understand this relationship.

In the general population, risk factors for OSA are age, male gender, body mass index (BMI), snoring, metabolic syndrome, and high blood pressure [15, 19–21]. Concerning alcohol consumption, smoking, benzodiazepines use, and Z-drugs use, data in the literature are contradictory about their potentially promoting effect in obstructive apneas [22–25]. Excessive daytime sleepiness is a common symptom in individuals with OSA [26] and may be measured by the use of the Epworth scale (ESS) [2]. However, its use as a predictor of OSA is controversial in the general population [27, 28]. Specifically, the severity of depression does not predict the presence of OSA [29] even though it is positively correlated with IAH [30]. Furthermore, in OSA, there are arguments that the presence of chronic systemic inflammation results in higher levels of C-reactive protein (CRP) [31]. In addition, OSA severity is correlated with the markers of this chronic inflammation [32, 33], but have never been studied as a predictor of OSA. Moreover, these various risk factors have never been studied for moderate to severe OSA in insomnia sufferers.

The aim of our study was to verify empirically the prevalence of moderate to severe OSA and to identify its risk factors with a large sample of insomnia sufferers to allow better detection and management of this syndrome in these individuals. The originality of our study is to focus mainly on moderate to severe OSA in insomnia sufferers, which is an under-studied and important public health issue.

## Methods

### Population

The sample was comprised of 1311 insomnia sufferers who were recruited retrospectively from the database of the general sleep laboratory of Erasme Hospital, which contains data for 3511 individuals who completed sleep laboratory monitoring in the years 2002–2014. This database includes information regarding a large range of primary sleep disorders and indications.

These insomnia sufferers were referred to the sleep laboratory by physicians specializing in sleep medicine after an ambulatory consultation during which a preliminary assessment of complaints related to sleep, ongoing treatments, somatic and psychiatric comorbidities was carried out systematically, making it possible to conduct a first diagnostic hypothesis. Following this first assessment, a sleep laboratory was offered to all insomnia sufferers to exclude the presence of other sleep pathology and obtain an objective evaluation of their sleep. Thus, only insomnia sufferers who accepted the sleep laboratory are present in the database. Moreover, the data obtained during this ambulatory consultation are systematically checked when the subjects are admitted to the sleep laboratory.

The inclusion criteria were age ≥ 18 years, the presence of diagnostic criteria A (complaint of difficulty initiating [defined as sleep latency ≥30 min at least 3×/week] or maintaining sleep [defined as 3 or more nocturnal awakenings or a long nighttime awakening (30 min or more) or early morning awakening (30 min or more before the usual time) at least 3×/week], or non-restorative sleep, for at least 1 month) and diagnostic criteria B (the sleep disturbance or associated daytime fatigue causes clinically significant distress or impairment in social, occupational, or other important areas of functioning) of insomnia from the Diagnostic and Statistical Manual of Mental Disorders fourth edition - Text Revision (DSM IV-TR) [34].

The exclusion criteria included the presence of a major psychiatric disorder (psychotic disorder), uncontrolled heavy somatic disease, chronic pulmonary disease, inflammatory or infectious disease, history of cranial trauma, history of central nervous system injury that could involve respiratory centres in the brain, history of craniofacial or thoracic cavity malformations, pregnancy, OSA already diagnosed or course of treatment before sleep laboratory, predominantly central apnea syndrome, narcolepsy or primary hypersomnia, parasomnia, and presence or history of substance abuse.

### Methods

#### Medical and psychiatric evaluation of participants

Upon admission to the sleep laboratory of Erasme Hospital, all subjects had their medical records reviewed and a complete somatic check-up performed, including a blood test, electrocardiogram, a daytime electroencephalogram, urinalysis, and a chest X-ray (only for those over the age of 45). These steps allowed for a systematic diagnosis of potential somatic pathologies present in people admitted to our unit.

Metabolic syndrome was diagnosed when three or more of the following criteria were fulfilled: fasting blood glucose ≥100 mg/dl or receiving treatment for diabetes mellitus, blood pressure ≥ 135/85 mmHg or receiving antihypertensive drug treatment, serum triglycerides ≥150 mg/dl, serum HDL-Cholesterol <40 mg/dl, or receiving treatment for dyslipidemia, and/or waist circumference ≥ 85 cm [35].

Patients also benefited on the day of admission from an appointment with a unit psychiatrist who potentially assigned psychiatric diagnoses per the DSM IV-TR criteria [34].

On admission, patients completed a series of self-questionnaires for an initial general assessment of their complaints as follows:

- The presence of depressive symptoms was investigated using the Beck Depression Inventory (BDI reduced to 13 items). This scale consists of 13 items that can be scored from 1 to 3. The final score can vary from 0 to 39. A final score of 0–4 indicates an absence of depression, 5–7 a slight depression, 8–15 a moderate depression, and >16 severe depression [36].
- Daytime sleepiness was investigated using the Epworth scale. This scale consists of eight questions that can be scored from 0 to 3 and assesses sleepiness during different daytime situations. The final score varies from 0 to 24. A final score greater than 10 indicates excessive daytime sleepiness [37].
- The presence of insomnia symptoms was investigated using the Insomnia Severity Index (ISI). This index consists of seven questions that can be scored from 0 to 4. The final score can vary from 0 to 28. A score of 0–7 indicates a lack of insomnia, 8–14 subclinical insomnia, 15–21 moderate insomnia, and 22–28 severe insomnia [38].

To avoid missing values, individuals who did not respond fully to these questionnaires were not included in our study.

### Sleep evaluation and study

A psychiatrist of the unit conducted a sleep-specific medical record systematic review on the day of admission to complete an assessment of complaints related to sleep including sleeping habits, symptoms of sleep apnea (snoring and self-reported apneas), symptoms of restless legs syndrome, and nocturnal movements (e.g., periodic movements of the limbs).

Participants stayed in a sleep laboratory for two nights, including a first night of habituation and a second night of polysomnography from which the data were collected for analysis. The patients went to bed between 22:00 - 24:00 and got up between 6:00 - 8:00, following their usual schedule. During bedtime hours, the subjects were recumbent and the lights were turned off. Daytime naps were not permitted.

The polysomnographic recordings from our unit met the guidelines of the American Academy of Sleep Medicine (AASM) [39]. The applied polysomnography-montage was as follows: two electro-oculogram channels, three electroencephalogram channels (Fz-Ax, Cz-Ax, and Oz-Ax, where Ax was a A1A2 mastoid reference), one submental electromyogram channel, electrocardiogram, thermistors to detect the oro-nasal airflow; finger pulse-oximetry, a microphone to record breathing sounds and snoring, piezoelectric sensors, strain gauges to measure thoracic and abdominal breathing, and leg movement electrodes. Polysomnographic recordings were visually scored by specialized technicians using AASM criteria [40] (inter-judge agreement score of 85%).

Apneas were scored if the decrease in airflow was ≥90% for at least 10 s, whereas hypopneas were scored if the decrease in airflow was ≥30% for at least 10 s with a decrease in oxygen saturation of 3% or followed by a micro-arousal [41]. AHI corresponds to the total number of apneas and hypopneas divided by period of sleep in hours. OSA was considered moderate to severe when AHI was ≥15/h [4].

### Statistical analyses

Statistical analyses were performed using Stata 14. The normal distribution of the data was verified using histograms, boxplots, and quantile-quantile plots; whereas, the equality of variances was checked using the Levene’s test.

Categorical data were described with percentages and numbers, and continuous data were described with means and SD or median and P25-P75. Normally distributed variables were analysed with a *t*-test. A Wilcoxon test or chi^2^ test were used on asymmetric distributed or dichotomous variables.

Univariate and multivariate binary logistic regression models were used to study the effects of risk factors on the occurrence of AHI ≥15. Risk factor variables included ESS score (categorical: ≤10, >10), BDI score (categorical: ≤8, >8 & <16, ≥16), ISI score (categorical: <15, ≥15 & <21, ≥21), BMI (categorical: <25 kg/m^2^, ≥25 & <30 kg/m^2^, ≥30 kg/m^2^), age (categorical: <50 years, ≥50 & <65 years, ≥65 years), CRP (categorical: ≤7 mg/L, >7 mg/L), and as binary variables for gender, snoring, metabolic syndrome, maintenance insomnia complaint, benzodiazepines use, Z-drugs use, antidepressants therapy, smoking, and alcohol consumption.

The automatic selection of risk factors in the model was performed by a stepwise backward method with an entry threshold of 0.05 and an exit threshold of 0.1. The adequacy of the model was verified by the Hosmer-Lemeshow test, whereas the specificity of model was verified by the Link test. Since the number of subjects by risk factors, outliers, and collinearity between risk factors may cause problems, they have also been verified.

A *p*-value of less than 0.05 was considered significant.

## Results

### Demographic data (Table 1)

Male gender, snoring, metabolic syndrome, alcohol consumption, and lower maintenance insomnia complaint are more frequent in subjects with AHI ≥15/h. These subjects also present an age/BMI/ESS score greater and BDI/ISI score lower than those with AHI <15/h. Markers of chronic inflammation, such as CRP, are higher in moderate to severe OSA. There was no significant difference in benzodiazepines use, Z-drugs use, antidepressants therapy, or smoking.

**Table 1:** Sample description (*n* = 1311)

Variables		Categories	%	Group AHI < 15/h	Group AHI ≥ 15/h	*P*-value Chi^2^
Gender		Male (*n* = 692)	52.78%	47.21%	87.36%	<0.001
		Female (*n* = 619)	47.22%	52.79%	12.64%	
Snoring		No (*n* = 646)	49.28%	54.30%	18.13%	<0.001
		Yes (*n* = 665)	50.72%	45.70%	81.87%	
Metabolic syndrome		No (*n* = 1199)	91.46%	93.98%	75.82%	<0.001
		Yes (*n* = 112)	8.54%	6.02%	24.18%	
Benzodiazepines use		No (*n* = 1080)	82.34%	82.02%	84.62%	0.394
		Yes (*n* = 231)	17.62%	17.98%	15.38%	
Z-drugs use		No (*n* = 1210)	92.30%	91.94%	94.51%	0.228
		Yes (*n* = 101)	7.70%	8.06%	5.49%	
Antidepressant therapy		No (*n* = 918)	70.02%	69.26%	74.73%	0.136
		Yes (*n* = 393)	29.98%	30.74%	25.27%	
Smoking		No (*n* = 1013)	77.27%	77.24%	77.47%	0.944
		Yes (*n* = 298)	22.73%	22.76%	22.53%	
Alcohol		No (*n* = 1010)	77.04%	78.74%	66.48%	<0.001
		Yes (*n* = 301)	22.96%	21.26%	33.52%	
Insomnia maintenance		No (*n* = 785)	59.88%	38.97%	47.25%	0.034
		Yes (*n* = 526)	40.12%	61.03%	52.75%	
	Mean ± SD					*t*-test
BMI (kg/m^2^)	26.92 ± 5.74			26.21 ± 5.47	31.31 ± 5.45	<0.001
		<25 (*n* = 570)	43.48%	49.07%	8.79%	
		≥25 & <30 (*n* = 411)	31.35%	30.56%	36.26%	
		≥30 (*n* = 330)	25.17%	20.37%	54.95%	
Age (years)	45.09 ± 12.41			43.78 ± 11.88	53.23 ± 12.55	<0.001
		<50 (*n* = 871)	66.44%	70.41%	41.75%	
		≥50 & <65 (*n* = 376)	28.68%	26.31%	43.41%	
		≥65 (*n* = 64)	4.88%	3.28%	14.84%	
	Median (P25-P75)					Wilcoxon test
CRP (mg/L)	1.5 (0.75 – 3.2)			1.4 (0.70 – 3)	2.1 (1 – 4.4)	<0.001
		≤7 (*n* = 1223)	93.29%	94.15%	87.91%	
		>7 (*n* = 88)	6.71%	5.85%	12.09%	
BDI	6 (3 – 11)			7 (3 – 11)	5 (3 – 9)	<0.001
		≤8 (*n* = 826)	63.01%	61.29%	73.62%	
		>8 & <16 (*n* = 327)	24.94%	25.51%	21.43%	
		≥16 (*n* = 158)	12.05%	13.20%	4.95%	
Epworth	10 (6 – 14)			6 (10 – 13)	11 (8 – 14)	<0.001
		≤10 (*n* = 713)	54.39%	56.33%	42.31%	
		>10 (*n* = 598)	45.61%	43.67%	57.69%	
ISI	18 (16 – 21)			19 (16 – 21)	18 (16 – 20)	<0.001
		<15 (*n* = 178)	13.58%	12.75%	18.68%	
		≥15 & <21 (*n* = 748)	57.06%	56.42%	60.99%	
		≥21 (*n* = 385)	29.37%	30.82%	20.33%	
AHI	1 (2 – 7)			2 (1 – 4)	30 (21 – 52)	<0.001
		<15/h (*n* = 1129)	86.12%			
		≥15/h (*n* = 182)	13.88%			

*BMI* Body Mass Index, *BDI* Beck Depression Inventory, *AHI* Apnea-Hypopnea Index, *ISI* Insomnia Severity Index

### Prevalence of OSA moderate to severe in major depression (Table 1)

The prevalence of OSA moderate to severe in our sample of 1311 insomnia sufferers was 13.88%.

### Univariate analysis (Table 2)

Male gender, snoring, ESS score > 10, BDI score ≤ 8, BDI score > 8 & <16, ISI score < 15, ISI score ≥ 15 & <21, metabolic syndrome, alcohol consumption, lower maintenance insomnia complaint, age ≥ 50 & <65 years, age ≥ 65 years, BMI ≥25 & <30 kg/m^2^, BMI >30 kg/m^2^, and CRP >7 mg/L were associated with an increased risk of moderate to severe OSA in insomnia sufferers.

**Table 2:** Univariate analysis (*n* = 1311)

Variables	% AHI <15/h	% AHI ≥15/h	OR (IC 95%)	*P*-value
Gender				<0.001
Male	77.02%	22.98%	7.73 (4.92 to 12.15)	
Female	96.28%	3.72%	1	
Snoring				<0.001
Yes	77.59%	22.41%	5.36 (3.61 to 7.96)	
No	94.89%	5.11%	1	
Epworth				<0.001
≤ 10	89.20%	10.80%	1	
>10	82.44%	17.56%	1.76 (1.28 to 2.41)	
BDI				0.002
≤ 8	83.78%	16.22%	3.21 (1.60 to 6.44)	
>8 & <16	88.07%	11.93%	2.24 (1.06 to 4.75)	
≥16	94.30%	5.70%	1	
ISI				0.004
< 15	80.90%	19.10%	2.22 (1.34 to 3.68)	
≥15 & <21	85.16%	14.84%	1.64 (1.10 to 2.43)	
≥21	90.39%	9.61%	1	
Metabolic syndrome				<0.001
Yes	60.71%	39.29%	4.97 (3.27 to 7.56)	
No	88.49%	11.51%	1	
Benzodiazepines use				0.394
Yes	87.88%	12.12%	0.83 (0.54 to 1.28)	
No	85.74%	14.26%	1	
Z-drugs use				0.231
Yes	90.10%	9.90%	0.66 (0.34 to 1.30)	
No	85.79%	14.21%	1	
Antidepressant therapy				0.137
Yes	88.30%	11.70%	0.76 (0.53 to 1.09)	
No	85.19%	14.81%	1	
Smoking				0.944
Yes	86.24%	13.76%	0.99 (0.68 to 1.43)	
No	86.08%	13.92%	1	
Alcohol				<0.001
Yes	79.73%	20.27%	1.87 (1.33 to 2.62)	
No	88.02%	11.98%	1	
Insomnia maintenance				0.035
Yes	87.77%	12.23%	1	
No	83.65%	16.35%	1.40 (1.02 to 1.92)	
BMI (kg/m^2^)				<0.001
< 25	97.19%	2.81%	1	
≥ 25 & <30	83.94%	16.06%	6.62 (3.77 to 11.62)	
≥30	69.70%	30.30%	15.05 (8.69 to 26.08)	
Age (years)				<0.001
< 50	91.27%	8.73%	1	
≥ 50 & <65	78.99%	21.01%	2.78 (1.98 to 3.92)	
≥ 65	57.81%	42.19%	7.63 (4.41 to 13.22)	
CRP (mg/L)				0.002
≤7	86.92%	13.08%	1	
>7	75.00%	25.00%	2.21 (1.33 to 3.69)	

*BMI* Body Mass Index, *BDI* Beck Depression Inventory, *AHI* Apnea-Hypopnea Index, *ISI* Insomnia Severity Index

### Multivariate analysis (Table 3)

In insomnia sufferers, risk factors obtained by the method of automatic selection (stepwise backward) that were associated significantly with an increased risk of moderate to severe OSA included male gender, snoring, lower maintenance insomnia complaint, ESS score > 10, metabolic syndrome, age ≥ 50 & <65 years, age ≥ 65 years, BMI ≥25 & <30 kg/m^2^, BMI >30 kg/m^2^, and CRP >7 mg/L.

**Table 3:** Summary of logistic regression for screening variables predicting AHI ≥15/h in insomnia sufferers (*n* = 1311)

Variables	Adjusted OR (IC 95%)	*P*-value
Gender		<0.001
Male	7.28 (4.41 to 12.00)	
Female	1	
Snoring		<0.001
Yes	2.59 (1.66 to 4.03)	
No	1	
Insomnia maintenance		0.026
Yes	1	
No	1.53 (1.05 to 2.22)	
Epworth		0.031
≤ 10	1	
>10	1.51 (1.04 to 2.21)	
CRP (mg/L)		0.022
≤ 7	1	
>7	2.12 (1.11 to 4.04)	
BMI (kg/m^2^)		<0.001
< 25	1	
≥ 25 & <30	3.36 (1.85 to 6.11)	
≥ 30	7.80 (4.27 to 14.26)	
Age (years)		<0.001
< 50	1	
≥ 50 & <65	2.05 (1.37 to 3.07)	
≥ 65	7.28 (3.68 to 14.40)	
Metabolic syndrome		0.025
Yes	1.79 (1.08 to 2.98)	
No	1	

Not included in the model because not significant: BDI, ISI, benzodiazepines, Z-drugs, antidepressants, smoking and alcohol

Adequacy of model: Hosmer-Lemeshow chi2 (*p* = 0.682)

Specificity of model: Linktest (linear component *p* < 0.001 and nonlinear component *p* = 0.233)

*BMI* Body Mass Index, *BDI* Beck Depression Inventory, *AHI* Apnea-Hypopnea Index, *ISI* Insomnia Severity Index

## Discussion

In our sample of insomnia sufferers, we demonstrated a prevalence of moderate to severe OSA of 13.88%, which is similar to that of the general population [14] but less than that of 29% highlighted in the literature [42]. However, in these studies, the sample of insomnia sufferers was relatively small and consisted only of subjects over 60 years of age. Since age is a known risk factor for OSA [15], this could result in greater recruitment of subjects with moderate to severe OSA in these studies and explain the difference in prevalence in our study. Furthermore, although the prevalence of moderate to severe OSA in insomnia sufferers appears to be similar to that of the general population and the co-occurrence of insomnia and OSA is very common [43, 44], the existence of an overlap between the symptoms of insomnia and OSA [42] may lead to the under-diagnosis of moderate to severe OSA in insomnia sufferers. These elements seem to indicate the necessity to globally manage these patients without focusing on a single complaint, such as insomnia, so as not to miss the diagnosis of moderate to severe OSA. Moreover, moderate to severe OSA is associated with increased cardiovascular morbidity and mortality [45, 46], justifying the need for implementation of effective treatments [47]. Therefore, in insomnia sufferers, it is important to identify the risk factors for moderate to severe OSA to allow better detection and management of this syndrome and reduce cardiovascular complications in these individuals.

In our study, as in the general population [15], male gender, snoring, age ≥ 50 years, and BMI ≥25 kg/m^2^ were identified as risk factors for moderate to severe OSA in insomnia sufferers. We therefore confirmed with a large sample of insomnia sufferers that the classical risk factors for moderate to severe OSA in the general population are applicable to insomnia sufferers. In the general population, there is a special relationship between OSA and metabolic syndrome. Indeed, subjects with a metabolic syndrome have a higher risk of severe OSA [21], whereas subjects with moderate to severe OSA have a higher risk of metabolic syndrome [48, 49]. In addition, the prevalence of metabolic syndrome increases with the severity of OSA [50]. However, in insomnia sufferers, no studies have investigated the relationship between OSA and metabolic syndrome, two frequent syndromes in insomnia sufferers [42, 51]. In our study, we demonstrated that, as in the general population, metabolic syndrome was also a risk factor for moderate to severe OSA in insomnia sufferers.

Despite two meta-analyses [27, 28], data in the literature concerning the use of excessive daytime sleepiness measured by ESS as a predictor of moderate to severe OSA in the general population are contradictory, and for insomnia sufferers, its use has not been studied. Yet, excessive daytime sleepiness is a risk factor of moderate to severe OSA in insomnia sufferers [52]. This element allows better understanding of why excessive daytime sleepiness measured by ESS is a risk factor of moderate to severe OSA in insomnia sufferers. Although some studies have shown a positive correlation between AHI and the severity of depression [30, 53], we have demonstrated similar to Ong et al. [29] that subjects with an AHI ≥15/h had a lower self-reported severity of depression than subjects with AHI <15/h, and that the self-reported severity of depression is not a risk factor for moderate to severe OSA. In addition, Bjorvatn et al. [54] have shown that the prevalence of insomnia decreased when the severity of OSA increased, which enables better understanding of our results. Indeed, in our study, we have shown that both the lower severity of insomnia (only in univariate analysis) and the lower complaint of maintenance insomnia are associated with a greater risk of moderate to severe OSA. This can be explained by the fact that in OSA, the fragmentation of sleep is characterized by the occurrence of micro-arousal [55], whereas in insomnia, it is marked by awakenings of longer duration [56]. Nevertheless, longer awakenings are clearly perceived in contrast to micro-arousal, which leads to greater complaints related to the maintenance of sleep. Therefore, in insomnia sufferers, excessive daytime sleepiness and lower maintenance insomnia complaints are risk factors for moderate to severe OSA, unlike the self-reported severity of depression.

In OSA, there are arguments in favour of chronic inflammation, which may be correlated with the severity of OSA [32, 33] and which may result in higher plasma levels of CRP [57]. Despite the special relationship between chronic inflammation and OSA, plasma CRP levels have never been studied as a risk factor for OSA in the general population or insomnia sufferers. However, we found that the presence of chronic inflammation in insomnia sufferers was a risk factor for moderate to severe OSA, which advances new perspectives in understanding the relationship between OSA and insomnia.

Although antidepressants may partially improve OSA by suppressing REM sleep and increasing upper airway tone [58], we demonstrated that antidepressants are not a risk or protector factor for moderate to severe OSA in insomnia sufferers. This can be explained by the fact that we did not distinguish between the different classes of antidepressants, which may possibly mask the protective or deleterious effect of certain molecules on respiration. Further, we found that benzodiazepines and Z-drugs are not risk factors for moderate to severe OSA, which seems to confirm the results of the meta-analysis of Mason et al. [25] However, we excluded subjects with dependence and therefore an overconsumption of these molecules. Benzodiazepines and Z-drugs are generally safe at low-doses for nocturnal breathing, but at high doses, they may cause or aggravate sleep apnea in more fragile patients [58]. Therefore, taking benzodiazepines and Z-drugs at a recommended dose in insomnia sufferers is not a risk factor for moderate to severe OSA.

The role of smoking in the occurrence of obstructive apnea is controversial in the literature [59, 60]. However, it would appear that nicotine would decrease the resistance of the upper airways with a consequent reduction of the risk of OSA, whereas in the case of withdrawal, this resistance would become more important and contribute to a greater risk of OSA [61]. However, no study has demonstrated a protective effect of smoking for OSA. In our study, we found that smoking is not a risk factor for moderate to severe OSA in insomnia sufferers. This may be explained by the fact that we included only active smokers who did not have nicotine withdrawal during their stay at the sleep laboratory. Similarly, in the literature, alcohol is a recognized risk factor for OSA. In fact, it induces a decrease in the tone of the upper airway muscles, which may increase the frequency and the severity of obstructive apnea in subjects with OSA, especially during the first hours of sleep [62]. In our study, we demonstrated that alcohol is not a risk factor for moderate to severe OSA in insomnia sufferers. This difference from the literature can be explained by the fact that none of the subjects included in our study had alcohol dependence and thus could stop without consequence their habitual consumption of alcohol during their stay at the sleep laboratory and avoid its deleterious effects on the nocturnal breathing.

In the future, prospective studies should be conducted with insomnia sufferers to validate the risk factors of moderate to severe OSA highlighted in our study. In addition, it would be useful to develop a score from these risk factors to better identify the insomnia sufferers at risk of moderate to severe OSA.

## Conclusion

In our study, we demonstrated with a large sample of insomnia sufferers that the prevalence of moderate to severe OSA was 13.88% and that the classical risk factors for moderate to severe OSA (male gender, age ≥ 50 & <65 years, age ≥ 65 years, BMI ≥25 & <30 kg/m^2^, BMI >30 kg/m^2^, and snoring) were applicable in insomnia sufferers. We also found that the presence of metabolic syndrome, excessive daytime sleepiness, lower maintenance insomnia complaint, and markers of chronic inflammation (CRP) were also risk factors for this syndrome in insomnia sufferers, unlike self-reported severity of depression, antidepressant therapy, smoking, alcohol consumption, and benzodiazepines and Z-drugs use. The identification of these different risk factors advances a new perspective for more effective screening of moderate to severe OSA in insomnia sufferers.

### Limitations

The results obtained in our study come from retrospective data that, even if they have been encoded in a systematic manner, cannot be verified directly with the subject, which means that our results need to be replicated in prospective studies. Moreover, we focused only on moderate to severe OSA, which means that our results cannot be generalized to other breathing disorders during sleep, such as central apnea syndrome. In addition, our database contains only insomnia sufferers who have agreed to perform a sleep laboratory, which may also limit the generalization of results.
